# Three‐dimensional printing CT‐derived objects with controllable radiopacity

**DOI:** 10.1002/acm2.12278

**Published:** 2018-02-07

**Authors:** Borhan Alhosseini Hamedani, Alexa Melvin, Kirubahara Vaheesan, Sameer Gadani, Keith Pereira, Andrew F. Hall

**Affiliations:** ^1^ Department of Mechanical Engineering College of Engineering Michigan State University 428 S. Shaw Lane East Lansing MI 48824 USA; ^2^ Department of Biomedical Engineering Parks College of Engineering, Aviation and Technology Saint Louis University 1 N. Grand Blvd. St. Louis MO 63103 USA; ^3^ Department of Radiology ‐ Interventional Radiology Saint Louis University School of Medicine 3635 Vista Blvd. St. Louis MO 63110 USA

**Keywords:** 3D printing, computed tomography phantoms, patient‐specific models, prostate artery embolization, radiopaque filaments

## Abstract

**Purpose:**

The goal of this work was to develop phantoms for the optimization of pre‐operative computed tomography (CT) scans of the prostate artery, which are used for embolization planning.

**Methods:**

Acrylonitrile butadiene styrene (ABS) pellets were doped with barium sulfate and extruded into filaments suitable for 3D printing on a fused deposition modeling (FDM) printer. Cylinder phantoms were created to evaluate radiopacity as a function of doping percentage. Small‐diameter tree phantoms were created to assess their composition and dimensional accuracy. A half‐pelvis phantom was created using clinical CT images, to assess the printer's control over cortical bone thickness and cancellous bone attenuation. CT‐derived prostate artery phantoms were created to simulate complex, contrast‐filled arteries.

**Results:**

A linear relationship (R = 0.998) was observed between barium sulfate added (0%–10% by weight), and radiopacity (−31 to 1454 Hounsfield Units [HU]). Micro‐CT scans showed even distribution of the particles, with air pockets comprising 0.36% by volume. The small vessels were found to be oversized by a consistent amount of 0.08 mm. Micro‐CT scans revealed that the phantoms' interiors were completely filled in. The maximum HU values of cortical bone in the phantom were lower than that of the filament, a result of CT image reconstruction. Creation of cancellous bone regions with lower HU values, using the printer's infill parameter, was successful. Direct volume renderings of the pelvis and prostate artery were similar to the clinical CT, with the exception that the surfaces of the phantom objects were not as smooth.

**Conclusions:**

It is possible to reliably create FDM 3D printer filaments with predictable radiopacity in a wide range of attenuation values, which can be used to print dimensionally accurate radiopaque objects derived from CT data. Phantoms of this type can be quickly and inexpensively developed to assess and optimize CT protocols for specific clinical applications.

## 1 INTRODUCTION

This work was motivated by a new Vascular and Interventional Radiology (VIR) procedure for the treatment of benign prostatic hyperplasia; Prostate Artery Embolization (PAE).^1^, ^2^ During the procedure, a microcatheter is navigated into bilateral prostatic arteries under x ray guidance. Embolization is then performed, causing the prostate gland to shrink over time, thus alleviating urinary symptoms and improving quality of life.^2^ PAE is technically challenging, even for experienced VIR physicians. This is mainly related to the prostatic arterial vascular anatomy where significant anatomic variants of prostate arteries exist, in terms of their origin, number, and course.^3^ In addition, the prostate arteries often have very small diameters, on the order of 0.5 to 1.5 mm.^4^

While C‐arm CT has sufficient spatial resolution to image these arteries, it is performed in the angiography lab *during* the procedure. Understanding the anatomy and planning the optimal C‐arm angles for device navigation *prior* to the embolization procedure has the potential to shorten procedures times, reduce cost, and reduce overall patient radiation exposure. A pre‐procedure CT angiogram (CTA) can therefore be very helpful in identifying these complex pathways. However, the inherently small prostatic arteries in some men are near the spatial resolution limit of most clinical CT scanners, and therefore may not be adequately visualized by a generic abdominal CTA protocol. The goal of the work presented here is to create patient‐specific complex anatomical phantoms for use in the CTA scanner protocol optimization for PAE.

Object visibility in CT is a function of both the size and contrast of the object in question.^5^ Commercial CT scanners have a multitude of data acquisition and image reconstruction parameters that affect the spatial resolution and contrast resolution of the resulting image. In addition, the contrast media injection protocol also affects object visibility, especially for the small arteries leading to the prostate. Visibility is also a function of the image visualization format; for example, in our experience, an artery may not be visible in a direct volume rendered (DVR) format and yet be visible in a maximum intensity projection (MIP) format.

While there are several commercial phantoms available to measure CT system parameters, they can be expensive and may not present the specific combination of attributes needed to optimize a CT data acquisition and image reconstruction protocol for a specific clinical scenario. For example, in the case of prostate artery imaging, the arteries are filled with some level of contrast, while embedded in soft tissue near the pelvis, which itself has a high level of attenuation.

Tissue‐equivalent materials, used in the construction of physical x ray phantoms, have been in development since the early 1900s.^6^ The first generation of physical phantom models were wax‐based, and included radiopaque fillers such as calcium carbonate, polyethylene, silicon dioxide, and titanium dioxide. More recently, epoxy resins have been used with the same additives,^7^ but also with phenolic microspheres to adjust the model density.^8^ Even before the advent of 3D printing, researchers used CT images to manufacture phantoms, making multi‐layered molds for collections of urethane‐based tissue‐mimicking materials.^9^ This prior work, however, differs from this study in that the investigators sought to mimic the xray properties of tissues at all x ray photon energies in the diagnostic spectrum using a combination of materials. Furthermore, this earlier work focused only on tissues (bone, lung and soft tissue) and not iodine‐filled blood vessels, as encountered in CTA.

3D printing anatomical structures from medical image data began in the early 1990's, and has been used primarily for three purposes: surgical planning, resident and patient education, and implantable prostheses.^10^ Cranio‐facial surgeons use stereolithographic models derived from CT scans for surgical planning.^11^ These models are used to show the relationship of complex anatomic structures that may not be fully appreciated when viewing a 2D image monitor, even for a DVR image. Surgical residents can attain a better understanding of pathological anatomy through the study of 3D anatomical models.^12^ Models can also be used to better explain complex surgical procedures to patients and their families. While early attempts at prosthesis printing were problematic, mainly due to the materials involved,^10^ this area nevertheless holds great promise for the future, as printing methods improve and material selections expand. 3D printing has also been used to create CT‐derived molds for tissue materials that cannot be printed, because of their nano‐scale structure,^13^ and to create hollow vascular structures which can be incorporated into flow phantoms.^12^, ^14^

Recently, there have been attempts to use various 3D printing technologies for constructing radiopaque objects. Jahnke et al. used a standard ink‐jet dot matrix printer filled with a potassium iodide solution to print radiopaque phantoms, derived from CT data, in several layers of printer paper which were then stacked to form 1 cm thick, three‐dimensional phantoms.^15^ This phantom type mimics lung, bone and soft tissue in aggregate only (e.g., in Hounsfield Unit (HU) values), as iodine has a K‐edge (33.2 keV) in the diagnostic x ray energy range.

Additive‐manufacturing printers buildup 3D objects layer by layer. Two varieties that have been used for CT phantom development are fused deposition modeling (FDM) printers (a generic technology with many manufacturers) and PolyJet printers (exclusive to Stratasys, Eden Prairie, MN). PolyJet printers deposit layers of liquid photopolymer which are instantly cured by ultraviolet light. While these printers can use only the manufacturer's polymers (which have not been developed for their x ray properties), they have been successfully used to create soft tissue CT phantoms.

To compare CT reconstruction algorithms, Solomon et al. developed a PolyJet 3D‐printed liver tissue phantom based on a texture model with parameters derived from CT images.^16^ They were able to achieve specific HU values using digital dithering of the available printer materials within a voxel, via custom software. The range of resulting HU values for the phantom was approximately 5 to 20 HU (at 120 kVp). Kiarashi et al. employed a PolyJet printer in the development of anthropomorphic breast phantoms, for evaluation of mammography and tomosynthesis equipment.^17^ While successful, a limited range of available printer material attenuation values resulted in the addition of filler materials to achieve the desired dynamic range. Leng et al. used a Polyjet printer to create CT‐derived liver and brain tissue phantoms.^18^ Attenuation values for these phantoms ranged from 70 to 121 HU (at 120 kVp), which again was restricted to the range of commercially available materials. To simulate contrast‐filled vessels in the liver, a dissolvable support material was used during the printing process. Once removed, this volume was filled with an iodine solution. In terms of cost, PolyJet systems start at $125,000, with material expenses ranging from $0.30/cm^3^ to $0.50/cm^3^.

FDM 3D printers use a solid filament which is melted, extruded through a nozzle (positioned by three stepper motors), and deposited onto the printed object where it adheres, quickly cools, and hardens. These printers are a potentially attractive option, due to their spatial resolution (< 1.0 mm) and the low cost of both the system (< $5,000) and the printer material (< $0.03/cm^3^). Unfortunately, while there are an increasing number of materials available for these printers, specific attention is not routinely paid to radiopacity. The two most common materials used in these printers, polylactic acid (PLA) and acrylonitrile butadiene styrene (ABS), have radiopacities lower than water.^19^ Ceh et al. were able to overcome this limitation using filaments that were manufactured by a third party, consisting of combinations of bismuth and ABS, to print both radiopaque 3D bone phantoms and x ray shielding equipment on an FDM printer.^20^ It is noteworthy that bismuth has a K‐edge at 90.5 keV, and therefore does not mimic tissue properties at specific x ray energies, but rather is only attenuating in aggregate. Madamesila et al. used an FDM printer and (commercially available) high impact polystyrene filament to fabricate low density phantoms for use in radiotherapy quality assurance applications.^21^ Variations in attenuation were achieved using the 3D printer's *infill percentage* parameter, which controls the percent of an object's inner volume that is filled with printer material, the remainder containing air. For this technique, mean attenuation values ranged from 0 to −800 HU. Similar work, using PLA, was performed by Oh et al.^22^

Our goal in prostate imaging is to develop a phantom, embedded in a water bath of human dimensions, which contains a pelvis and an iliac artery tree, to use in optimizing the CTA imaging protocol. The phantom will be constructed such that iliac artery trees of various shape, dimension and opacity may be inserted into the phantom. The purpose of the work presented here is to develop a reliable and inexpensive method of manufacturing CT phantoms by extruding 3D printer filaments composed of varying mixtures of a standard FDM 3D printer material and a radiopaque doping agent, and then using those filaments to produce patient‐specific CT phantoms of the pelvis and iliac artery tree, with controlled radiopacity.

## 2 MATERIALS AND METHODS

### 2.A Printer filament development

#### 2.A.1 Material selection

Two doping agents were evaluated: calcium carbonate and barium sulfate. Calcium carbonate has a long history of use in bone phantoms.^7^, ^8^ Barium sulfate has a K‐edge at 37.4 keV, and therefore has not been used for bone. However, its K‐edge is close to that of iodine (33.1 keV), making it a reasonable material to simulate iodine‐filled blood vessels. The potential problem in using calcium carbonate is the volume of material required to achieve the radiopacity of bone, relative to the base material. To evaluate this issue, a mixture of 20% calcium carbonate powder and 80% ABS plastic, by weight, was extruded. Mineral oil was added to facilitate adherence of the calcium carbonate to the ABS pellets. For this mixture, 15% of the calcium carbonate did not adhere to the ABS pellets and was left at the bottom of the container. In our experience, powder that is not adhered to the pellets will settle at the bottom of the extruder. Test objects printed with this filament had an attenuation of approximately 65 HU. Furthermore, a micro‐CT scan revealed a significant number of air pockets. We therefore concluded that it is not possible to 3D print material with a radiopacity approximating cortical bone (~1000 HU) using calcium carbonate and this filament creation technique.

Using the NIST XCOM website,^23^ it was determined that the linear attenuation coefficient of barium sulfate at 50 keV is more than 25 times higher than that of calcium carbonate. Since barium sulfate would require much less material to achieve a similar radiopacity, we chose to proceed with using this doping agent for all phantoms, including both the iliac artery and pelvis phantoms.

Two materials were evaluated as the base material for the extruded filaments: PLA and ABS. Pellets of both material types, doped with 5% barium sulfate by weight, were extruded in a Filabot Original Filament Extruder (Filabot, Barre, VT). While an exhaustive evaluation was not performed, the ABS pellets yielded more consistent results in terms of filament extrusion, print reliability, and object durability. With PLA we had difficulty achieving a large and consistent diameter, there were more print failures, and the resulting prints were more fragile. We therefore proceeded using ABS.

#### 2.A.2 Filament creation

Five filament types were created from mixtures of ABS pellets (IC3D industries, Columbus, OH) and barium sulfate powder (Sigma Aldrich, St. Louis, MO), in the proportions listed in Table 1. Mineral oil was added to the ABS pellets to facilitate barium sulfate adhesion during the mixing process. The barium sulfate was first crushed with a mortar and pestle, and then added to the ABS/oil mixture and further mixed until the pellets were evenly coated.

**Table 1:** Quantities mixed, by weight, for 3D printer filament extrusion

BaSO_4_ (% weight)	BaSO_4_ (g)	ABS (g)	Mineral oil (g)
0.0	0.00	50.00	0.00
2.5	1.25	48.75	0.07
5.0	2.50	47.50	0.12
7.5	3.75	46.25	0.17
10.0	5.00	45.00	0.24

The filament was extruded in 50 g batches using the Filabot filament extruder, set to an extrusion temperature of 185°C, with an extrusion die diameter of 2.54 mm. Because the filament diameter takes some time to stabilize, undoped ABS was used initially, with doped ABS added once a stable diameter was achieved. The extruded filament was fed to a Filabot Spooler (Filabot, Barre, VT), placed 25 cm from the extrusion die exit. After extrusion, the undoped filament portion was removed, and the remaining section was measured with a digital caliper every 10 cm. The average was computed and used as the filament diameter printing parameter.

### 2.B Phantoms printed and scanned

Four types of phantoms were printed using the barium sulfate doped filaments. First, simple cylinder phantoms were printed to assess the HU values as a function of barium sulfate added. Next, complex artery tree phantoms were designed and printed to assess the accuracy of the printer in printing cylindrical segments of small diameter. Third, a pelvis was printed to develop and assess a method to print large multi‐piece objects derived from CT data, and to assess the use of the *infill percentage* parameter to produce an object with an inner core (representing cancellous bone) that had a lower HU value than the outer shell (representing cortical bone). Finally, a CT‐derived iliac artery tree, including the prostate artery, was printed to assess the system's ability to print complex vessel anatomy using the radiopaque filament.

The phantoms were printed on Lulzbot TAZ 5 and TAZ 6 FDM printers (Aleph Objects, Inc., Loveland, CO). Cura software (Lulzbot version 21.04, Ultimaker, Netherlands) converted the stereolithograph files defining each phantom into G‐code (printer commands), and was also used to set the printing parameters. The *filament diameter* parameter allows the printer to adjust the feed rate of the filament to achieve a consistent volumetric extrusion rate from the nozzle. This was set equal to the measured average filament diameter (defined above) of the filament segment created. The remaining printer parameters used are listed in Table 2. The patient CT data in this work was used under an IRB waiver from Saint Louis University (SLU) Hospital. All phantom CT scans were performed in a water bath of human dimensions, on a Siemens Definition Flash CT Scanner (Siemens Healthcare, Erlangen, Germany). The tube potential was 100 kVp, and the CARE dose setting resulted in exposure values of approximately 300 mAs; parameters which are consistent with clinical CT scans of the pelvis and prostate arteries. The slice thickness and slice spacing were both 0.6 mm. The image size was 512 × 512 pixels, with a pixel size of 0.78 mm. The reconstruction filter was B20f. Micro‐CT scans were performed on a μCT35 system (Scanco Medical, Wayne, PA) with tube potential of 70 kVp, and an exposure of 34 μAs. Images were reconstructed at an isotropic resolution of 10 microns.

**Table 2:** 3D printer parameters for each object printed

Printer parameter	Cylinder	Artery tree	Pelvis	Prostate artery
Filament diameter (mm)	Meas. Avg.^a^	Meas. Avg.	Meas. Avg.	Meas. Avg.
Infill percentage (%)	100	100	10 & 20	100
Shell thickness (mm)	1.0	1.0	1.0 & 2.0	1.0
Infill top and bottom (Y/N)	YES	YES	NO	YES
Layer height (mm)	0.25	0.25	0.25	0.25
Printer speed (mm/s)	50	50	50	50
Nozzle temperature (°C)	230	230	230	230
Bed temperature (°C)	110	110	110	110
Nozzle size (mm)	0.5	0.5	0.5	0.5

a Meas. Avg. is the average of the filament diameter measurements.

#### 2.B.1 Cylinder phantoms

A cylinder phantom with a diameter of 1.0 cm and a height of 1.0 cm was CAD designed (3DS Max Design 2015, Autodesk, San Rafael, CA) and exported into a stereolithography (STL) file. Two batches of each filament type were extruded, and four cylinders were printed from each batch, resulting in a total of eight cylinders per filament type. The cylinders were printed using an infill percentage of 100%. To evaluate the content of the cylinders prior to scanning, density measurements were taken using the immersion method described in ISO 1183‐1.^24^ In this method, an object's mass is measured both in air and while suspended in a water bath that is not sitting on the scale. Using these two measurements, the density is obtained as:

(1) $$\rho =\frac{m_{a}}{m_{a}-m_{w}}$$

where ρ is the specimen's density, *m*_*a*_ is the mass measured in air, and *m*_*w*_ is the mass measured with the object suspended in water. This was then compared with the theoretical density of the mixture, computed as:

(2) $$\rho =\frac{1}{\frac{m_{\mathrm{BS}}}{\rho_{\mathrm{BS}}}+\frac{m_{\mathrm{ABS}}}{\rho_{\mathrm{ABS}}}}$$

Where *m*_*BS*_ and *m*_*ABS*_ are the fractional masses of barium sulfate and ABS, respectively, and ρ_*BS*_ and ρ_*ABS*_ are the densities of pure barium sulfate and pure ABS, respectively. The cylinders were then fixed on suitable frames which were then mounted in a custom‐made water bath (Planet Plexi Corp, Laguna Hills, CA). The x‐y dimensions of the water bath (40 cm × 25 cm with semi‐circular sides) were selected based on the averaged pelvic‐area outer body dimensions from seven SLU Hospital patient CT scans. The water‐filled test object was then scanned in the Siemens CT scanner as described above. To examine the homogeneity of barium sulfate particle distribution within the cylinders, and the possible presence of air pockets, a micro‐CT scan of representative sections of each cylinder type was also performed.

#### 2.B.2 Artery tree phantom

**Figure 1:**
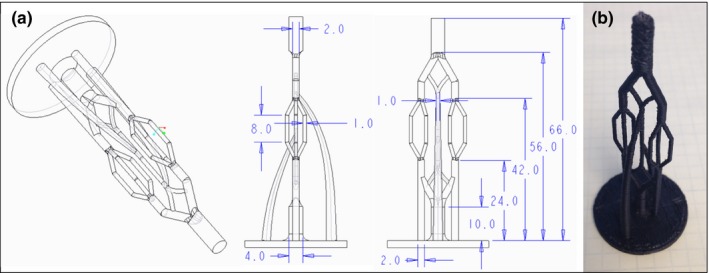
(a) CAD drawing and (b) photograph of the artery tree model used to measure the dimensional accuracy and printability of small diameter objects printed on the 3D printer.

To evaluate the ability of the printer to produce completely filled, small‐diameter segments, an artery tree phantom was CAD‐designed (Creo V2, PTC, Needham, MA), containing segment diameters of 1.0, 2.0, and 4.0 mm (Fig. 1). The printer had a nozzle diameter of 0.5 mm, and it was questionable whether a 1.0 mm diameter branch could be printed correctly with this printer and these types of filaments. Three phantoms were printed (with 2.5%, 5% and 10% doped filaments) using an infill percentage of 100%. Ten measurements, at arbitrary locations on each phantom, for each tree segment diameter, were taken with a digital caliper, for a total of 30 measurements for each diameter. Micro‐CT scans of representative sections of one phantom were taken to evaluate the internal make‐up of the phantom, including air pockets.

#### 2.B.3 Pelvis phantom

A phantom, derived from a CT scan, of the left half of a human pelvis was constructed. The pelvis is composed of a dense cortical bone shell, with an inside of spongy cancellous bone. The 3D printer is capable of manufacturing structures with both a programmable shell thickness and a programmable infill percentage. These printing parameters were recruited to demonstrate the ability of the printer to print bone models with variable cortical bone thickness and variable cancellous bone radiopacity. The phantom was designed such that the cortical bone would be considered the shell of the 3D printed object (filled completely with filament), and the cancellous bone would be considered the inside of the object (filled with a filament support grid). When filled with water, the cancellous bone radiopacity of the phantom would be a function of the filament HU value and the infill percentage, computed as:

(3) $$HU_{cancellous\;\;bone}=infill\times HU_{\mathrm{filament}}+\left(1-infill\right)\times HU_{\mathrm{water}}$$

where *infill* is the 3D printer infill percentage, *HU*_*filament*_ is the HU value of the filament, and *HU*_*water*_ is the HU value of water (= 0 HU).

**Figure 2:**
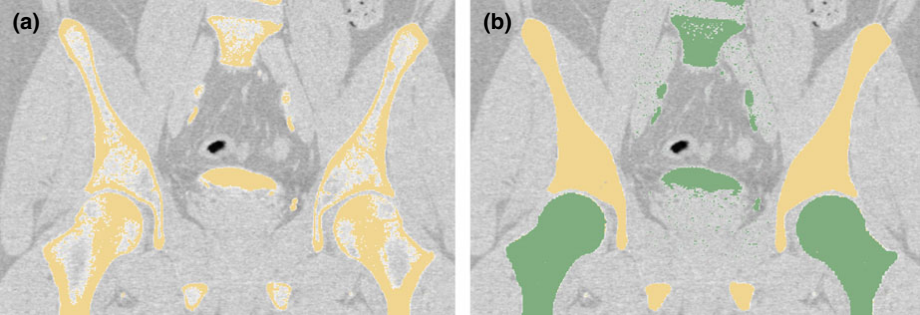
Human CT image, shown in the 3D slicer software package, used to create the pelvis model. (a) Initial threshold‐based segmentation (yellow). (b) Segmentation after the pelvis (yellow) has been separated from the remaining bones (green) and the cancellous bone sections filled in.

**Figure 3:**
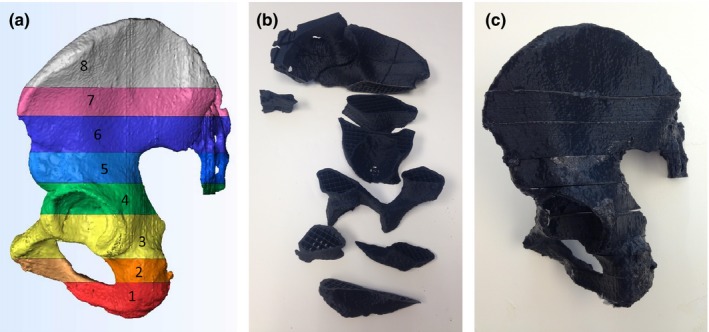
CT‐derived half‐pelvis model shown as (a) CAD‐based model cut into 8 components for 3D printing, (b) 3D printed components (partially assembled and flipped to show reverse side), and (c) fully assembled half‐pelvis model.

The phantom was based on a SLU Hospital patient CT scan. First, the pelvis bone was manually separated from surrounding tissues including the femurs, vertebra, and soft tissues using the open‐source 3D Slicer software package (http://www.slicer.org)^25^ as depicted in Fig. 2. 3‐matic software (Research Version 11.0, Materialise, Leuven, Belgium) was then used to divide the model into smaller, printable pieces, as presented in Fig. 3(a).

The components making up the left half of the pelvis and sacrum were printed with filament consisting of 6.0% barium sulfate. This percent was chosen by measuring the average maximum HU value from 10 cortical bone locations of one slice of the human pelvis CT (930 ± 156 HU) and using a regression line (Fig. 6) computed from the cylinder measurements (which yielded 6.08%, with 6.0% predicting 918 HU). While cortical bone thickness in the patient image ranged from less than 1.0 mm to over 5.0 mm, a uniform shell thickness of 2.0 mm was chosen to demonstrate the ability of the printer to successfully deviate from the default parameter of 1.0 mm. The infill percentage was set to 20%. This was chosen by averaging ten sample areas (region of interest = 38 mm^2^) of cancellous bone from the same slice of human pelvis as above (193 ± 51 HU) and using this measurement in Eq. (3), which yielded 20.8%. There was one exception to these parameters. Segment 8 [as shown in Fig. 3(a)] was printed with a shell thickness of 1.0 mm and an infill percentage of 10%. The parts were printed with no top or bottom (a printer parameter) so that they could be filled with water.

The individual parts of the left pelvis were submerged in the water bath where any remaining air was removed using water from a small hose. The parts were attached at the edges by emerging them slightly from the water bath and fusing them with a soldering iron. The individual segments, as well as the integrated model, are shown in Figs. 3(b) and 3(c). The phantom, within the water bath, was then scanned.

#### 2.B.4 Prostate artery phantom

**Figure 4:**
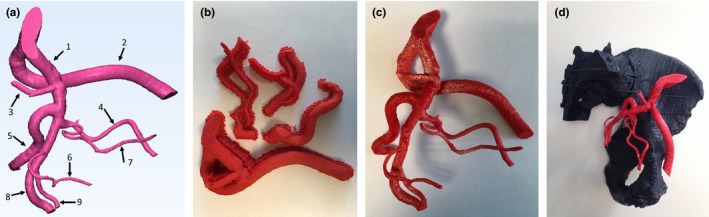
Iliac artery tree as shown (a) segmented in Mimics, (b) 3D‐printed as components with support material, (c) fully assembled model, and (d) attached to the pelvis phantom. Arteries printed include (1) internal iliac, (2) external iliac, (3) lateral sacral, (4) obturator, (5) superior gluteal, (6) inferior vesical, (7) prostate, (8) inferior gluteal, and (9) pudendal.

An anonymized human pelvic CTA dataset was used to render and print a radiopaque left iliac artery tree, including the prostate artery. Mimics software (Research Version 19.0, Materialise, Leuven, Belgium) was used to separate the arteries from the remainder of the image, resulting in the artery tree shown in Fig. 4(a). The resulting model was then separated into four components which were exported as STL files. Two complete models were printed; one with filament containing a barium sulfate percentage of 2.5% and the other with 3.7% filament. These percentages correspond to predicted radiopacities of 365 HU and 555 HU, respectively, and are in the range observed for contrast‐enhanced iliac arteries in clinical CTA scans. The support material [Fig. 4(b)] was removed and the components were assembled using ABS glue (consisting of ABS and acetone). The four artery segments (which make‐up one iliac artery tree model) with their support material and the completed phantom are shown in Figs. 4(b) and 4(c). Finally, each iliac artery tree was sequentially attached to the pelvis model [Fig. 4(d)], placed in the water bath and scanned.

## 3 RESULTS

### 3.A Extruded filament

**Figure 5:**
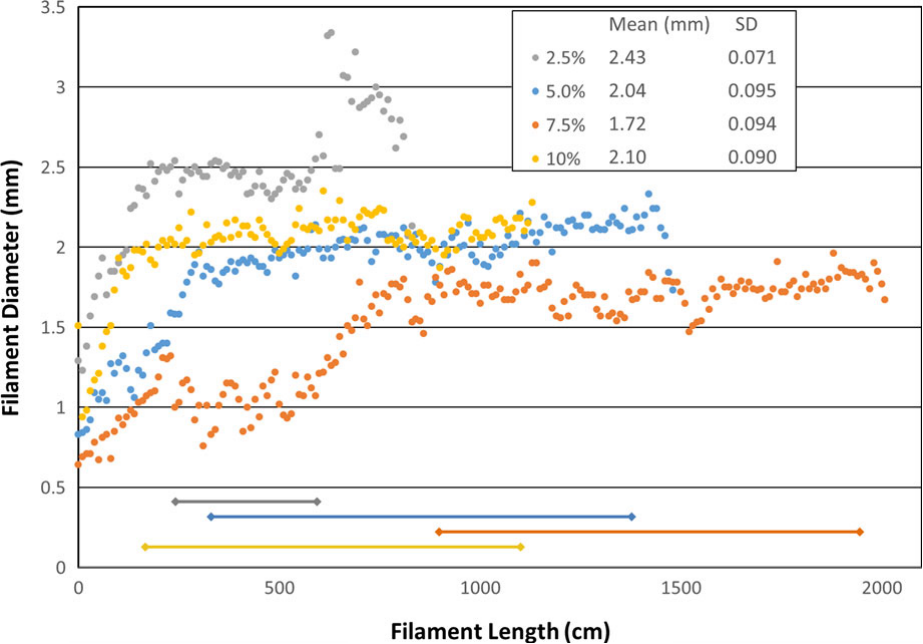
Measured filament diameters (in 10 cm intervals) along the length of an extruded 3D printer filament segment, for four types of filament extruded (excluding undoped). The line segments at the bottom indicate the filament regions over which the mean and standard deviation were computed. Note that the larger the filament diameter, the shorter the overall segment length.

Filaments of all doping levels were successfully extruded. Representative results of the filament diameters and lengths are shown in Fig. 5. The resulting filaments ranged in average diameter (in their regions of consistent diameter) from 1.72 to 2.43 mm with standard deviations ranging from 0.071 to 0.095 mm. The standard deviation for one commercially produced 2.85 mm filament was measured to be 0.021 mm. Contiguous filament segment lengths ranged from 3 to 10 m. Shorter segment lengths corresponded to larger filament diameters. Shorter segment lengths did not affect the printing of larger objects (e.g., the pelvis components) as the printer is able to pause for the insertion of a new filament segment, and then seamlessly continue.

### 3.B Printed phantoms

#### 3.B.1 Cylinder phantoms

Once printed, the density of each of the 40 cylinders was measured. Density measurements were able to identify one cylinder that was not consistent because of its low barium sulfate content. This difference was confirmed by the CT scan. It was found that this filament came from a segment too close to the beginning of the extrusion, where the filament still contained undoped barium sulfate pellets. This cylinder was not included in the analysis.

**Figure 6:**
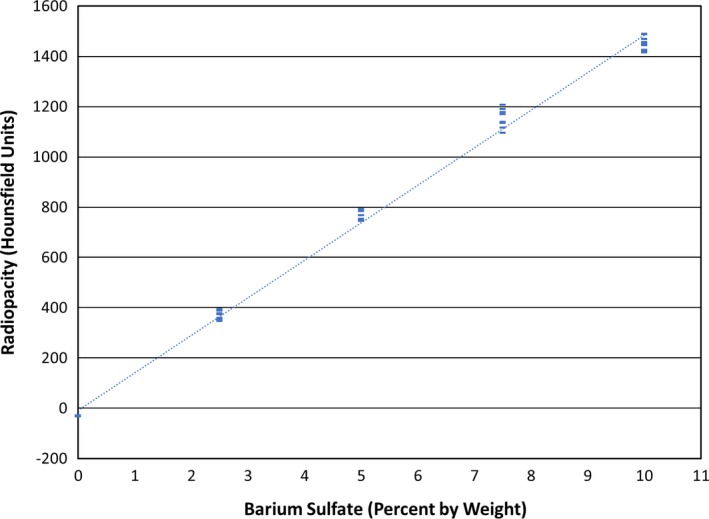
3D‐printed cylinder radiopacity as a function of barium sulfate percentage, by weight, along with the associated regression line. The mean value for each printed cylinder is shown. The Pearson correlation coefficient is 0.998 for all samples, and the regression line formula is HU = 149 × (BS%)−8.5. The regression line formula used to compute the barium sulfate percentages for the pelvis and iliac artery phantoms excluded the 10% samples and is: HU = 158 × (BS%)−30.

The remaining cylinder images were characterized using an open‐source image analysis software package (ImageJ 1.49v, NIH, Bethesda, MD) over a volume of five consecutive slices. For each slice, the mean and variance were computed for a 22 mm^2^ region‐of‐interest, placed at the center of the circular cylinder image. A mean and composite standard deviation (the square root of the sum of the variances) for the five‐slice volume was then computed. The mean HU value for each cylinder is plotted in Fig. 6, and the average means and average standard deviations are shown in Table 3. The Pearson correlation coefficient for all of the means was 0.998.

**Table 3:** Measurement statistics for cylinder models used to examine radiopacity

BaSO_4_ (weight %)	Average mean (HU)^a^	Average SD (HU)
0.0	−31	22
2.5	366	25
5.0	764	27
7.5	1153	36
10.0	1454^b^	135

a Average mean is the mean of the average HU values measured for each of the eight cylinders.

b Seven cylinders were measured.

**Figure 7:**
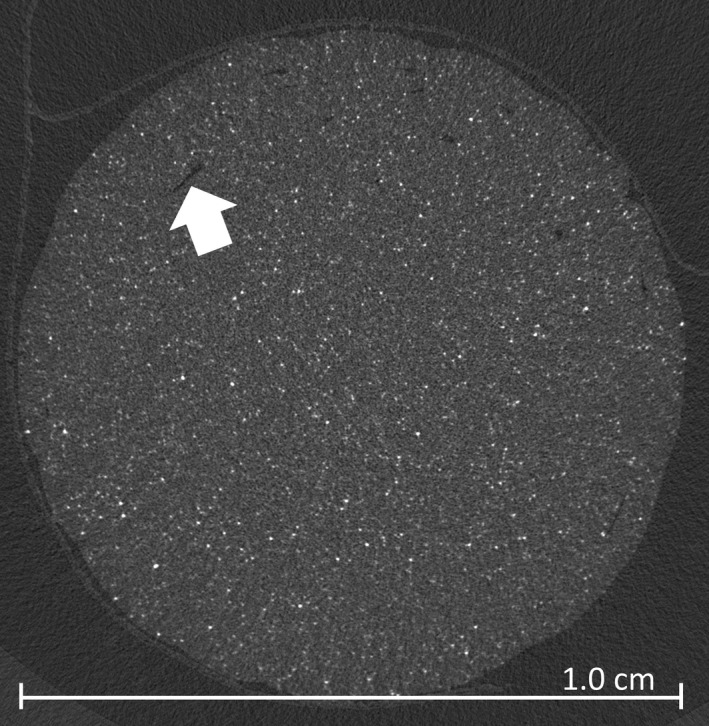
Micro‐CT image of a 5% doped, 1.0 cm cylinder model. Small air pockets are evident (arrow) but infrequent. HU value measurement of ten sampled regions (2.5 mm diameter) yielded a standard deviation that was 5.7% of the average mean.

Micro‐CT images were analyzed to assess both particle distribution and the presence of air pockets. Upon visual inspection of the images, as shown in Fig. 7 for a 5% doped cylinder, the distribution of barium sulfate particles appeared even, and there were few artifacts resulting from the printing process. One example artifact (showing a seam between the shell and area of infill) is evident in Fig 7. For one slice of the 5% doped cylinder, the means of ten 5 mm^2^ regions were measured, using ImageJ, to assess particle distribution throughout the slice. The mean and standard deviation of this measurement was 1374 ± 89 HU.

Five consecutive micro‐CT slices were analyzed using ImageJ, to estimate the volume of air pockets in the samples. After smoothing, negative values were categorized as air. (Noise reduction helped delineate air from ABS, and 0 was the center point between means in a bi‐modal image histogram in a small region of interest.) The average volume of air measured was 0.36%.

#### 3.B.2 Artery tree phantom

**Figure 8:**
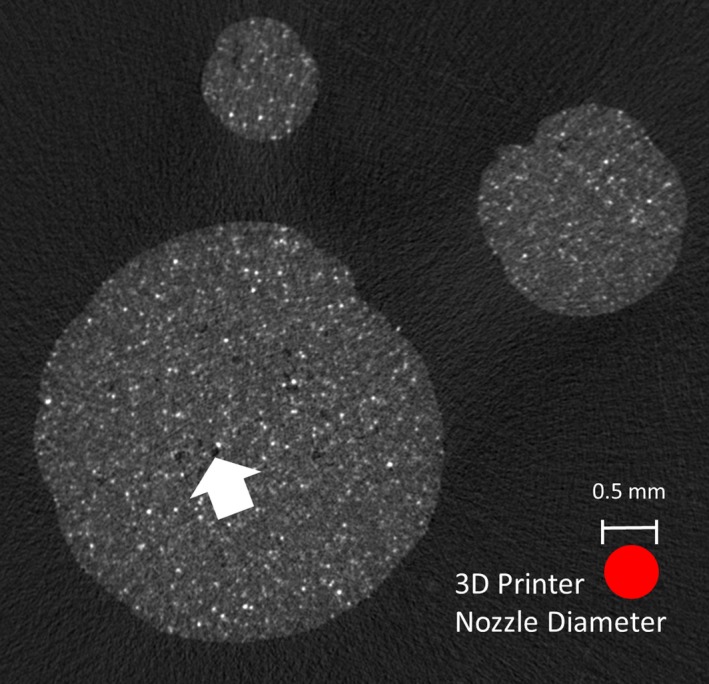
Micro‐CT image of three 3D‐printed vessel segments of varying diameters: 1.0 mm, 2.0 mm and 4.0 mm. The printer nozzle diameter was 0.5 mm, as shown. Note that small air pockets (arrow) are visible but infrequent showing that the 3D printer can reliably print and fill small‐diameter structures.

Results of the dimensional measurements of the artery tree models are shown in Table 4. All measured diameters were slightly larger than the CAD‐specified diameter, by an average of 0.08 mm. A micro‐CT image of representative sections of each artery diameter is presented in Fig. 8. Another purpose of the scan was to evaluate the ability of the 3D printer to completely fill segments with small diameters. Even though the printer nozzle diameter (0.5 mm) is close to the diameter of the vessel segments, the printer was able to completely fill in the area of all three segment types, with minimal air pockets and minimal printer‐induced artifact.

**Table 4:** Measurement statistics for artery tree phantoms

Specified diameter (mm)	Mean measured diameter (mm)	SD	SD as % of Mean	Minimum measured diameter (mm)	Maximum measured diameter (mm)
1.00	1.071	0.038	3.55	1.01	1.14
2.00	2.089	0.051	2.44	2.00	2.18
4.00	4.087	0.052	1.27	4.00	4.19

#### 3.B.3 Pelvis phantom

**Figure 9:**
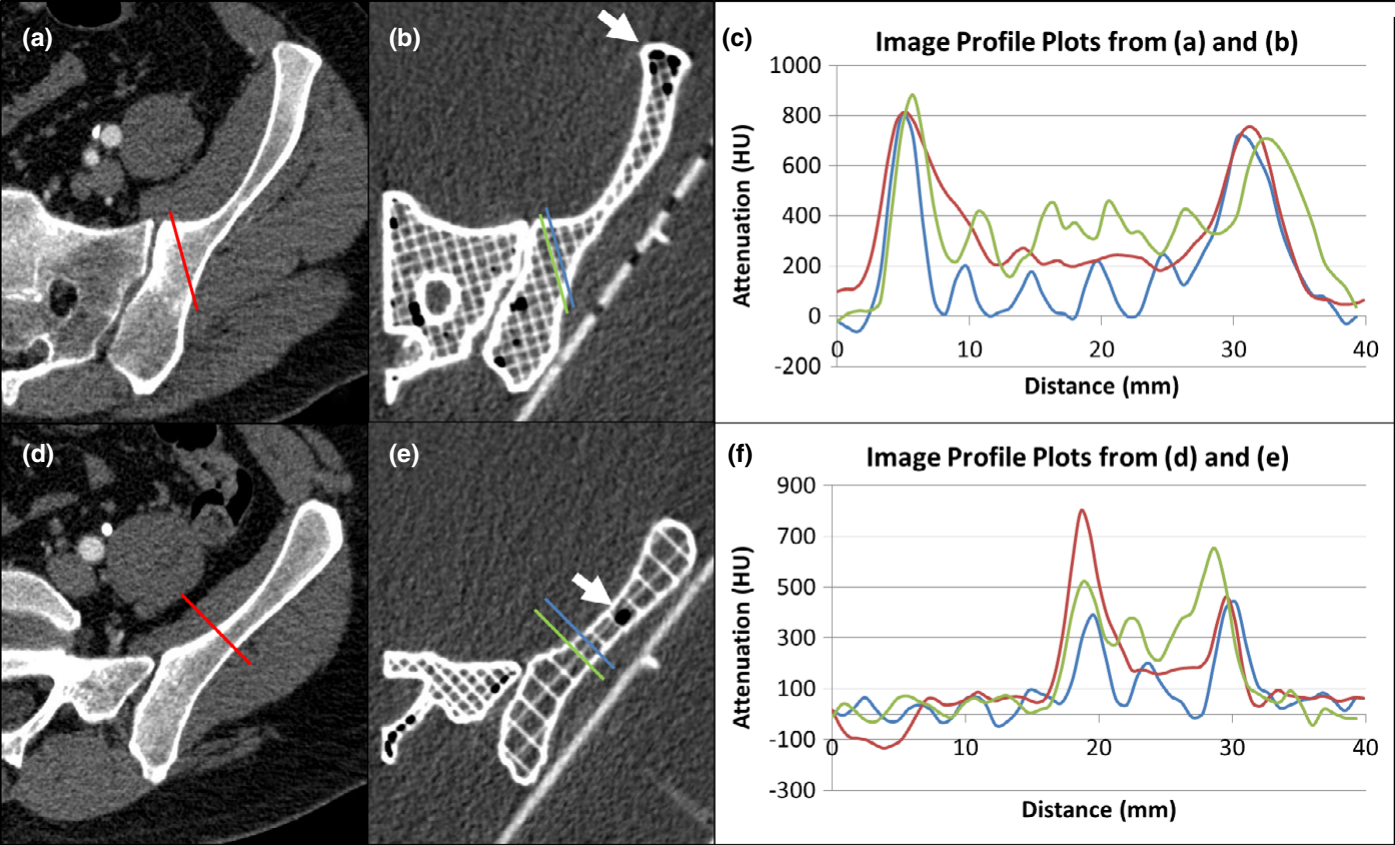
Two representative slices of a CT image of the 3D‐printed pelvis model, (b) and (e), and the corresponding clinical CT image slices, (a) and (d). The profile plots in (c) and (f) show the clinical and phantom image attenuation for both cortical and cancellous bone sections. Data locations are indicated by lines of matching color in the images, with 0 mm corresponding to the upper left end‐point of each line. Note that an area average is required to compute the average cancellous bone attenuation in the phantom, as the attenuation is less homogenous than the clinical image. Air pockets are visible (arrows).

Two representative axial slices of the pelvis phantom, one with an infill percentage of 20% (from component 7) and one with an infill percentage of 10% (from component 8), are shown in Fig. 9 along with the corresponding human source images. For each phantom slice, ten samples of maximum cortical bone HU values [as can be observed in Figs. 9(c) and 9(f)] were averaged, yielding 714 HU and 476 HU, respectively. The average measured HU value for a 1 cm diameter cylinder printed from the same 6% doped filament was 944 HU. Also for each slice, ten 50 mm^2^ regions of cancellous bone were averaged. The resulting averages were 200 HU and 131 HU for the 20% and 10% infill percentages, respectively. The average attenuation for the clinical cancellous bone sample, used to specify the 20% infill parameter, was 193 HU. These data are summarized in Table 5. Matching example profile plots were made for one region of each slice, shown in Figs. 9(c) and 9(f). Two profile plots were made for each phantom: one crossing a water‐filled section, and one crossing an infilled section.

**Table 5:** Summary of parameters used in the construction of, and measured in the evaluation of, the pelvis phantom

Bone Type	CT image mean Attn (HU)^a^	BaSO_4_% via regression (Fig. 6)	BaSO_4_% used in extrusion and print	Predicted Attn (HU)	Measured test cylinder Attn (HU)	Measured average maximum Attn in phantom (HU)
Cortical	930	6.08	6.00	918	944	2 mm: 714 1 mm: 476
**Bone Type**	**CT image mean Attn (HU)**	**Infill % via Eq.** **(3)**	**Infill % used in extrusion and print**	**Predicted Attn (HU)**		**Measured average Attn in phantom (HU)**
Cancellous	193	20.8	20.0	20% Infill: 184 10% Infill: 92		20% Infill: 200 10% Infill: 131

a Attn is attenuation in HU.

The filament segments required to make the phantom totaled 49 m in length, had an extrusion time of 9 h, and cost $9.80. The total print time was 16 h.

#### 3.B.4 Prostate artery phantom

**Figure 10:**
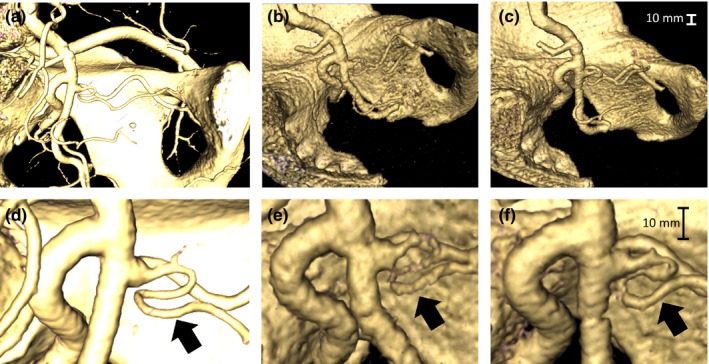
DVR visualizations of a CT image of a human pelvis and iliac artery tree including the prostate artery (arrows) (a and d), and CT images of 3D‐printed models with iliac arteries printed from filaments with radiopacities of 365 HU (b and e), and 555 HU (c and f). Note the more glossy appearance of the human pelvis as compared to the rough surface of the pelvis model. The DVR settings for b and e are such that the speckle noise from the water bath is just visible. The pelvis and prostate artery images were taken from two different patients.

The CT scans of the two prostate artery phantoms (365 HU and 555 HU, each scanned with the pelvis phantom in a water bath) were evaluated in both axial‐planar and DVR formats. Mean attenuation was measured in 10 consecutive axial slices (region‐of‐interest = 22 mm^2^) of the inferior iliac artery (i.e., a larger artery) of each scan. The resulting means and standard deviations were 365 ± 12 HU and 557 ± 9 HU, respectively. The DVR results, rendered using 3D Slicer, are shown in Fig. 10 along with the clinical image containing the original prostate artery. DVR visualization is a particularly effective method for determining both the origin (parent vessel) and configuration (e.g., multiple ostia) of the prostate artery. One clear difference between the human CT image and the model CT images is the surface of both the pelvis and arteries, which is smoother in the human data, and therefore reflects light differently. For both models, the DVR threshold setting (controlling the scalar color and scalar opacity maps) was adjusted to optimize visualization of the PA. In Figs. 10(b) and 10(e), showing the 365 HU artery tree, background noise from the water bath is just visible as speckle in the background of the image. However, the artery is not optimally visualized. Due to a combination of the spatial resolution of the CT scanner and the image reconstruction filter, the HU value for this segment of the model was less than 200 HU. Similarly, the artery's HU value for the 555 HU model, shown in Figs. 10(c) and 10(f) was less than 400 HU. However, this HU level allowed better visualization of the prostate artery.

## 4 DISCUSSION

This work has demonstrated the feasibility of creating radiopaque 3D printer filaments of controllable and consistent radiopacity and using those filaments in an FDM printer to create inexpensive CT phantoms derived from CT image data. Achievable HU values with doped ABS‐based filaments ranged from −31 to 1454 HU, corresponding to barium sulfate percentages, by weight, of 0 to 10%. There was a strong linear correlation between barium sulfate percentage and radiopacity (R = 0.998). However, for the highest percentage filament (10%), this correlation deviated slightly, as slightly lower values of radiopacity were measured. A probable cause for such a deviation is the limited surface area of the ABS pellets, to which the barium sulfate is able to adhere. This phenomenon is consistent with our experiments with calcium carbonate, which proved unsuitable for use with this filament creation technique.

The most difficult parameter to control in extrusion was filament diameter consistency. The extruder produced filament with a standard deviation that was approximately four times greater than commercial (undoped) filament. The Cura printer control software has a filament diameter parameter that is used to compute a constant volumetric extrusion rate at the nozzle. However, it cannot be changed during the printing process, and any variations in filament diameter will result in the extrusion of too much or too little material. This can lead to either bulges or air gaps in the shell or infill of an object. Anecdotally, we found that objects printed with commercial filament were slightly more consistent than those printed with our extruded filament. It seems clear that the process presented here would benefit from a commercially extruded filament. The quality assurance steps of measuring object density and measuring filament diameter were critical to achieving predictable results.

Micro‐CT images revealed an even distribution of the radiopaque agent within the phantoms, with only very small air pockets or other artifacts of the FDM printing process, as shown in Figs. 7 and 8. While vessel segments as small as 1.0 mm could be reliably printed, there was a small bias in the diameter (+0.08 mm). This seems to be the result of printing with a 100% infill percentage. The printer manufacturer recommends a 70% infill percentage even for load‐bearing parts, and states that using a 100% infill percentage will result in oversized objects.^26^ This infill percentage was selected to maximize the x ray attenuation, and to investigate the homogeneity of the extruded filaments more easily, as the air content adds complexity to the calculations and experiments. Furthermore, this gives a truer representation of contrast filled arteries. It would be easy to compensate for this oversizing effect by decreasing the artery size accordingly in the CAD file, or by increasing the threshold slightly when segmenting the medical image file.

The design and construction of the pelvis phantom demonstrated the ability to 3D print a human‐scale radiopaque bone phantom from CT image data. Furthermore, this work demonstrated the ability of the printer to be configured to vary the cortical bone thickness, via the shell thickness parameter, as well as vary the cancellous bone attenuation (as a percent of the cortical bone attenuation) via the infill percentage parameter. This was accomplished using the same image‐derived stereolithography file, setting these parameters at print time.

The attenuation measurements of the cortical bone sections of the phantom, both for the 1.0 mm and 2.0 mm shell thickness settings (476 HU and 714 HU), varied substantially from the target value (918 HU), even though a test cylinder printed from the same filament had an average attenuation of 944 HU. The attenuation profiles in Figs. 9(c) and 9(f) appear to be a low‐pass filtered version of the actual phantom profile. This could be due to a combination of partial‐volume effects and spatial filtering performed during image reconstruction. It would be possible to choose, either with iteration or inverse filtering, a filament attenuation value that would yield results that were closer to the clinical value. This difference in HU values also reveals the potential value in these types of phantoms. For example, because the human CT image had undergone similar image formation and processing effects, one could conclude that the cortical bone attenuation in this image is not an accurate representation. This would be an interesting area for further work.

The average attenuation of the cancellous bone section of the phantom component with 20% infill (200 HU) compared very well to the target value derived from the clinical image (184 HU). These measurements were average values (rather than the peak value measured for cortical bone), and any spatial filtering would not affect the average value (i.e., zero spatial frequency). The attenuation value for the component with 10% infill (131 HU) had a larger difference from the target value (92 HU) than the 20% sample. One possible cause could be variations in the extruded filament diameter, as this affects the volumetric flow rate at the printer nozzle, which is used in the infill percentage calculation.^26^ In contrast to Madamesila et al.,^21^ we used only two different infill percentages, as opposed to their twenty, and so did not perform a complete characterization of this parameter. An investigation of a greater variety of infill percentages using small CAD‐based phantoms would better characterize this relationship. It should also be noted that the documented infill percentage is slightly higher than the actual infill percentage, as the software does not account for the overlap in the crosshatching pattern [as shown in Fig 9(b)].^26^

We filled the phantom with water to reach clinically relevant attenuation values in the cancellous bone sections with a lower infill percentage (and therefore less filament) than would have been required for air. However, it was difficult to entirely eliminate the air from the phantom, as shown in Figs. 9(b) and 9(e). Care was taken to divide the pelvis into sections for which there would be a minimum of completely enclosed spaces, and the top and bottom of each phantom were not printed to allow water access. However, during the attachment process, the edges of each section had to be raised out of the water slightly to be fused, allowing some air to return. Future experiments will include the use of marine grade epoxy for attachment. One limitation of this phantom is that the material is not a true tissue‐mimicking material for bone. Because of the K‐edge in barium sulfate x ray attenuation, it does not have the linear attenuation coefficient of tissue at each of the photon energies emitted by a clinical CT scanner x ray tube. Calcium carbonate doping could provide such a property, however, we were not able to achieve usable attenuation levels with this material and our technique.

The attenuation of the prostate arteries was also affected by the CT scanning and image formation process. The smaller arteries had lower attenuation than the larger arteries, clearly the result of some combination of partial volume effects and reconstruction filtering. Future work will seek to determine which scanning and reconstruction protocols minimize this difference, and also how these protocols affect artery visibility. These phantoms could also be used to evaluate contrast delivery. We have observed lower HU values in the prostate arteries in clinical scans, relative to those in the iliac arteries. The question is whether this is a processing artifact, as described above, or a problem with the contrast timing. This phantom could be used to determine the HU value for a small prostate artery when there is consistent contrast filling throughout the iliac artery tree, which could then be compared to clinical CT scans to potentially detect a suboptimal contrast algorithm: a combination of contrast amount, injection rate, and scan delay.

As shown in Fig. 10, the surface texture of the models produced in this work did not match that of the anatomy, as imaged by the CT scanner and visualized by the DVR technique. While 3D objects are built in discrete layers in FDM printers, we do not believe this is the cause of the issue. The printer layer height parameter of 0.25 mm would induce artifacts of a much higher spatial frequency. The difference is more likely the result of the image segmentation technique, which could be addressed in two ways. It is possible to smooth a segmented image with Mimics prior to STL file export. This effect could be assessed prior to export by examining the edges of the segmented model. It is also possible to smooth the printed object by brushing it with acetone. As medical image interpretation is subjective, we believe this is an important issue to address going forward.

While Polyjet printers have higher spatial resolution and a wider range of materials overall, they are much more expensive and are currently not able to achieve the attenuation values required for bone and contrast‐filled artery phantoms. The capital equipment cost for this work was $5100.00 and the object printing cost, including ABS pellets and barium sulfate, was $0.03/cm^3^. Finding a commercial maker of these filaments would make printing the phantoms easier and more reliable, and would reduce construction time by more than one‐third.

Future work includes printing a variety of CT‐derived prostate arteries in a range of radiopacities, for use in the optimization of CT scanner acquisition and reconstruction settings for optimal DVR visualization of the prostate artery. This will provide a better understanding of the specific tradeoffs between contrast dose, radiation dose and image quality for this application. More reliable and higher quality imaging of these arteries will also enable clinicians to better plan the embolization procedure, including device selection and C‐arm angulations, which should lead to reduced procedure times and procedure cost. It may also be possible to identify patients who are not candidates for the procedure, due to excessive vessel tortuosity.

In addition to PAE, there are other innovative procedures, such as Bariatric Embolization, that could benefit from this approach to pre‐procedure CT image optimization, as VIR physicians continue to pursue more targeted intra‐vascular therapies. These phantoms can indeed be applied to the development of any new CT imaging protocol where the anatomical complexity may not be well represented by more general purpose phantoms. CT protocols are often developed iteratively, in patients. Much of this iteration could potentially be avoided. Finally, these tools could be used to better interpret the results of a specific CT scan, as exemplified by the cortical bone attenuation in the pelvis phantom.

## CONFLICT OF INTEREST

No conflicts of interest.
